# Hypovitaminosis D in persons with Down syndrome and autism spectrum disorder

**DOI:** 10.1186/s11689-023-09503-y

**Published:** 2023-10-25

**Authors:** Natalie K. Boyd, Julia Nguyen, Mellad M. Khoshnood, Timothy Jiang, Lina Nguyen, Lorena Mendez, Noemi A. Spinazzi, Melanie A. Manning, Michael S. Rafii, Jonathan D. Santoro

**Affiliations:** 1 Division of Neurology, Children’s Hospital Los Angeles, 4650 Sunset Blvd, MS82, Los Angeles, CA 90027 USA; 2 Princeton University, Princeton, NJ USA; 3 Department of Pediatrics, Benioff Children’s Hospital, University of California San Francisco, Oakland, CA USA; 4 Department of Genetics, Stanford University School of Medicine, Palo Alto, CA USA; 5 Department of Neurology, Keck School of Medicine of the University of Southern California, Los Angeles, CA USA; 6 Alzheimer’s Therapeutic Research Institute, University of Southern California, San Diego, CA USA

**Keywords:** Down syndrome, Autism spectrum disorder, Vitamin D 25-OH, Immunity, Autoimmune, Neurodevelopmental, Trisomy 21

## Abstract

**Background:**

Plasma levels of vitamin D have been reported to be low in persons with Down syndrome (DS) and existing data is limited to small and homogenous cohorts. This is of particular importance in persons with DS given the high rates of autoimmune disease in this population and the known relationship between vitamin D and immune function. This study sought to investigate vitamin D status in a multi-center cohort of individuals with DS and compare them to individuals with autism spectrum disorder (ASD) and neurotypical (NT) controls.

**Methods:**

A retrospective, multi-center review was performed. The three sites were located at latitudes of 42.361145, 37.44466, and 34.05349. Patients were identified by the International Classification of Diseases (ICD)-9 or ICD-10 codes for DS, ASD, or well-child check visits for NT individuals. The first vitamin D 25-OH level recorded in the electronic medical record (EMR) was used in this study as it was felt to be the most reflective of a natural and non-supplemented state. Vitamin D 25-OH levels below 30 ng/mL were considered deficient.

**Results:**

In total, 1624 individuals with DS, 5208 with ASD, and 30,775 NT controls were identified. Individuals with DS had the lowest mean level of vitamin D 25-OH at 20.67 ng/mL, compared to those with ASD (23.48 ng/mL) and NT controls (29.20 ng/mL) (*p* < 0.001, 95% CI: −8.97 to −6.44). A total of 399 (24.6%) individuals with DS were considered vitamin D deficient compared to 1472 (28.3%) with ASD and 12,397 (40.3%) NT controls (*p* < 0.001, 95% CI: −5.43 to −2.36). Individuals with DS with higher body mass index (BMI) were found to be more likely to have lower levels of vitamin D (*p* < 0.001, 95% CI: −0.3849 to −0.1509). Additionally, having both DS and a neurologic diagnosis increased the likelihood of having lower vitamin D levels (*p* < 0.001, 95% CI: −5.02 to −1.28). Individuals with DS and autoimmune disease were much more likely to have lower vitamin D levels (*p* < 0.001, 95% CI: −6.22 to −1.55). Similarly, a history of autoimmunity in a first-degree relative also increased the likelihood of having lower levels of vitamin D in persons with DS (*p* = 0.01, 95% CI: −2.45 to −0.63).

**Conclusions:**

Individuals with DS were noted to have hypovitaminosis D in comparison to individuals with ASD and NT controls. Associations between vitamin D deficiency and high BMI, personal autoimmunity, and familial autoimmunity were present in individuals with DS.

**Supplementary Information:**

The online version contains supplementary material available at 10.1186/s11689-023-09503-y.

## Introduction

Down syndrome (DS) is the most common genetic cause of intellectual disability, occurring in roughly one in 700 live births [1, 2]. Individuals with DS have a variety of co-occurring conditions that can occur across the lifespan, with autoimmune diseases such as Hashimoto’s thyroiditis, celiac disease, and type one diabetes being highly prevalent [3, 4].

Vitamin D has been reported to be low in persons with DS, but nearly all of these studies come from small and homogenous cohorts [5–7]. In addition, individuals with DS and associated autoimmune disease or obesity have been noted to have particularly high rates of hypovitaminosis D [5]. Vitamin D has been linked to the modulation of T-cell proliferation [8, 9], cytokine modulation [10], and protection against the development of a variety of autoimmune diseases [11] and has become increasingly viewed as an important contributor to autoimmune disorders. Given the high rate of autoimmune disorders observed in this population, knowledge of vitamin D status could provide an opportunity for potential therapeutic interventions.

The cause of hypovitaminosis D in individuals with DS remains unknown although other neurodevelopmental disorders, such as autism spectrum disorder (ASD), have reports of similar findings [12, 13]. In addition, there does appear to be a similar inflammatory signal to hypovitaminosis D in this population as well [14]. Although much more genetically diverse, individuals with ASD have some similar neurological and medical co-morbidities to individuals with DS which serves as a similar comparator group. Research on the role of vitamin D supplementation in this population is more advanced than in the DS literature although the results of such interventions have been mixed [15, 16].

The purpose of this study was to assess vitamin D status in a cohort of individuals with DS and compare them to individuals with ASD and a control group without a neurodevelopmental disorder diagnosis (neurotypical (NT) control). Furthermore, we investigated if there was an association between vitamin D status and the presence of autoimmune disease and neurological disorders in persons with DS.

## Methods

### Approvals and study setting

Following individual Institutional Review Board approvals at each site, a multi-center, retrospective chart review was performed. The three clinical sites were Boston, MA; Palo Alto, CA; and Los Angeles, CA, all located in the USA. All sites were tertiary academic medical centers with dedicated centers for both DS and ASD.

### Data availability

Anonymous data for this study is available on reasonable request to qualified researchers following approval and authorization of the IRBs.

### Patient identification

Electronic medical records (EMRs) were utilized to identify patients. A query of individuals with either an International Classification of Diseases (ICD)-9 (758.0) or ICD-10 (Q90) code for DS was performed. From the study population, any patient with at least one vitamin D 25-OH (25(OH)D) level was included. The control population was obtained by querying any patient with a 25(OH)D level performed in the outpatient setting of the main clinical site’s health system. From this group, patients with ASD were isolated as a separate patient population and were identified by either an ICD-9 (299.0 and/or 299.01) or ICD-10 (F84.0 and/or F84.5) code for ASD. Neurotypical controls were identified by using any of the following ICD-9: V20.2, V20.31, V320.32, V70, V70.5, V70.6, V73 or ICD-10 codes: Z00.0, Z00.129, Z00.11, Z00.8, Z01.0, Z01.4, Z01.81, Z02.89, Z71.84, or Z13.3 and no other associated codes. These were all codes corresponding to well-child care (Supplement 1). The date range for this search within the medical record was 01/01/2000–06/01/2019.

### Inclusion/exclusion criteria and groups

Three groups of patients were analyzed in this study: individuals with DS, individuals with ASD, and controls without either DS or ASD and no other chronic medical or neurodevelopmental condition. All patients had to be aged birth to 26 years and have at least one 25(OH)D level recorded. Individuals with DS and/or ASD were excluded if they had a history of neoplasia, regardless of chemotherapy or radiation status. This was determined by reviewing co-occurring ICD-9 or ICD-10 codes listed in the medical record. Controls were age- and sex-matched and were excluded if they had any ICD-9 or ICD-10 code outside of the ones listed for their screening within the medical record. Supplementation of vitamin D2 or D3 was not exclusionary for involvement in this cohort as supplementation status was unknown or unreported in nearly all records.

### Data extraction

The charts of all individuals with DS were manually reviewed. Vitamin D supplementation status and compliance were not readily available or improperly documented in nearly all patients and thus were not utilized. Individuals with ASD and individuals in the control group had demographic and biometric data extracted, but other information was not readily available. The month that the 25(OH)D level was obtained was recorded as well to assess for seasonal variation.

### Vitamin D measures, multiple recordings, and definition of deficiency

Cholecalciferol (vitamin D3) and ergocalciferol (vitamin D2) are both metabolized by the liver into 25(OH)D which is measured in serum to determine a person’s vitamin D status. As 25(OH)D levels reflect dietary and ultraviolet sources of vitamin D, it is one of the most used measures of vitamin D status assessment.

For this study, the first 25(OH)D level recorded in the EMRs was used as it was felt to be the most reflective of a natural and non-supplemented state. This was significant as supplementation data was not available. All 25(OH)D levels extracted were continuous variables between 0 and 200 ng/mL. Levels of 25(OH)D below 30 ng/mL were considered deficient per American Academy of Pediatrics guidelines [17].

### Statistical analysis

Descriptive statistics were produced for demographic and clinical presentations. Means and standard deviations were calculated for continuous variables and proportions for categorical variables. Chi-squared analysis was performed to evaluate the differences between the sub-groups. Multivariable linear regression models were applied to investigate the association between 25(OH)D levels with patient demographics, site of recruitment, and clinical characteristics. The adjusted mean difference in 25(OH)D level per unit increase in a patient characteristic was estimated along with the corresponding 95% confidence interval. A *p* nominal value of < 0.05 was considered statistically significant for each statistical test.

## Results

In total, 37,607 individuals were identified as having at least one vitamin D 25-OH level obtained, including 1624 with DS, 5208 with ASD, and 30,775 controls. In the neurotypical control group, 94% (*n* = 28,928) of visits were coded as either Z00.129 (encounter for routine child health examination), Z00.8 (encounter for other general examination), or Z00.0 (encounter for general adult medical examination). Demographic data is reported in Table 1. Overall, there were 18,920 (50.4%) male participants and 18,687 (49.6%) female participants with no statistically significant differences between groups (*p* = 0.14, 95% CI: 0.78–2.36). The mean age across all three groups was 13.9 years (standard deviation ± 9.8 years). All three groups had a majority of Caucasian participants, notably making up a significantly larger percentage in the DS cohort (83.31%) than in the ASD group and the control group (40.8% and 41.7%, respectively) (*p* < 0.001, 95% CI: 0.34–0.78). There were also more individuals with DS of Hispanic ethnicity (59.05%) than in the ASD and NT cohorts (41.8% and 42.8%, respectively) *(p* < 0.001, 95% CI: 0.33–0.88)*.* Patient volume was consistent across the three sites with no statistically significant variance (*p* = 0.21, 95% CI: 0.59–2.04).

**Table 1:** Demographic and vitamin D status in children with Down syndrome, autism spectrum disorder, and controls

	Down syndrome (*n* = 1624)	Autism spectrum disorder (*n* = 5208)	Neurotypical controls (*n* = 30,775)	*p* value	95% CI
Age (mean, SD)	11.5 (4.73)	12.1 (5.57)	15.1 (4.02)	0.07	0.63–1.03
Sex					
*Male*	788 (48.5%)	2668 (51.2%)	15,464 (50.3%)	0.14	0.78–2.36
*Female*	836 (51.5%)	2540 (48.8%)	15,311 (49.7%)		
Race					
*Caucasian*	1353 (83.3%)	2145 (40.8%)	12,818 (41.7%)	<0.001	2.97–9.64
*Asian*	159 (9.8%)	381 (7.3%)	2022 (6.6%)		
*Black*	85 (5.23%)	135 (2.6%)	816 (2.65%)		
*Nat. Amer*	10 (0.62%)	0 (0%)	0 (0%)		
*Other*	14 (0.86%)	1549 (29.7%)	9262 (30.1%)		
*Unknown*	3 (0.18%)	1018 (19.5%)	5857 (19.3%)		
Ethnicity					
*Hispanic*	959 (59.1%)	2174 (41.7%)	13,175 (42.8%)	<0.001	1.37–5.21
*Not Hispanic*	665 (40.9%)	1822 (34.9%)	10,640 (34.6%)		
*Unknown*	0 (0%)	1212 (23.3%)	6960 (22.6%)		
Site of recruitment					
*Boston, MA*	534 (33%)	1691 (32%)	9813 (32%)	0.21	0.59–2.04
*Palo Alto, CA*	489 (30%)	1927 (37%)	10,923 (36%)		
*Los Angeles, CA*	601 (37%)	1590 (31%)	10,019 (33%)		
BMI (mean, SD)	23 (8.5)	19.33 (8.1)	19.3 (7.9)	<0.001	−0.38 to −0.15
Vitamin D 25-OH (mean, SD)	20.67 (11)	23.48 (11.8)	29.20 (22)	<0.001	−8.97 to −6.44
Vitamin D deficient (*n*, %)	399 (24.6%)	1472 (28.3%)	12,397 (40.3%)	<0.001	−5.43 to −2.36

*Nat. Ameri* Native American, *CI* Confidence interval, *SD* Standard deviation

**Fig. 1:**
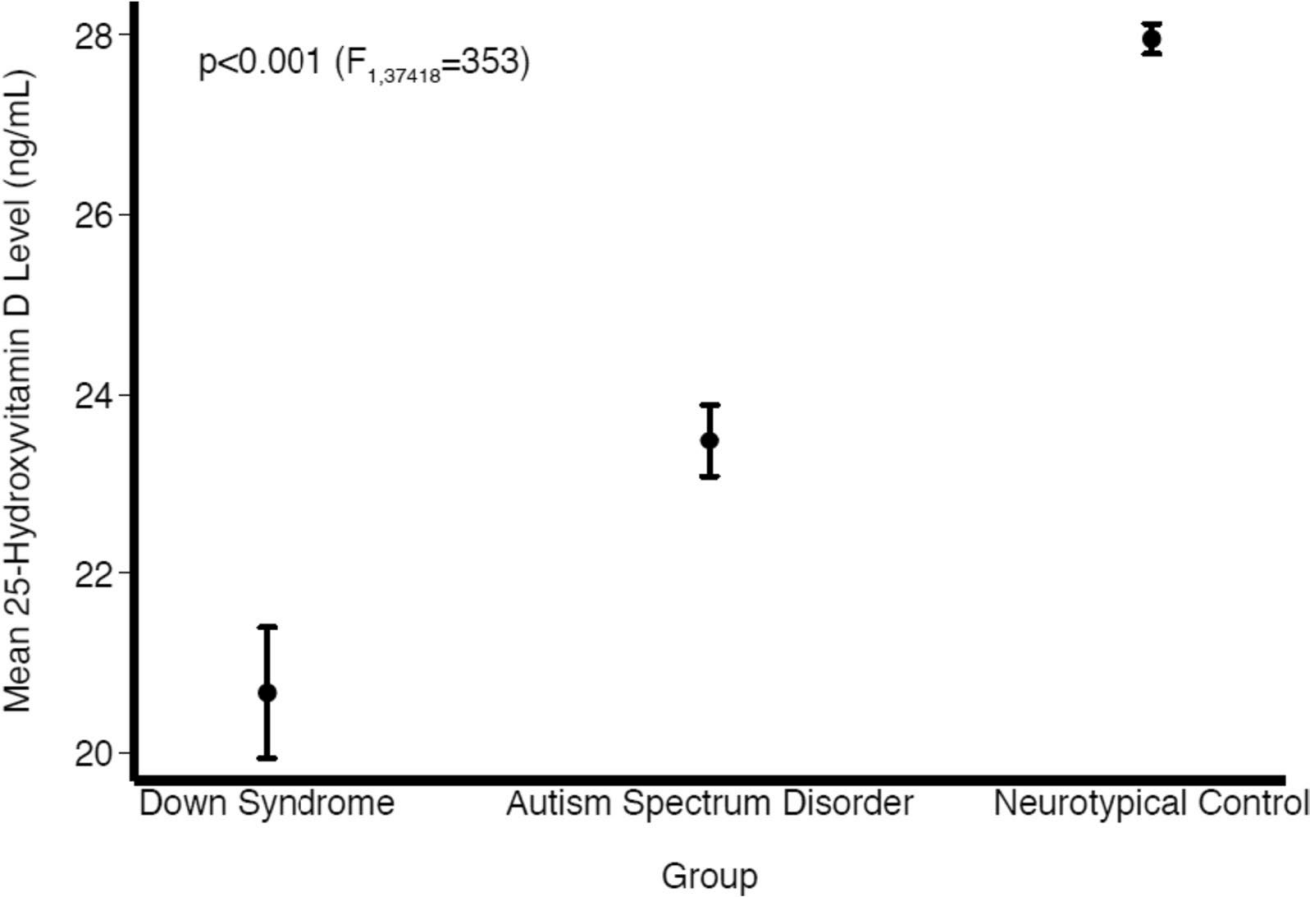
Mean 25(OH)D level by group. Legend: Mean 25(OH)D levels across the three groups. The one-way ANOVA *F*-test value is presented along with the corresponding *p* values. The confidence intervals (bars) illustrate the range of potential values of the mean 25(OH)D levels and allow comparisons of mean levels between any two groups which are all statistically significantly different from one another

Vitamin D status by study group is reported in Table 1 and Fig. 1. Individuals with DS had the lowest mean level of 25(OH)D (20.67 ng/mL) compared to those with ASD (23.48 ng/mL) and NT controls (29.20 ng/mL) (*p* < 0.001, 95% CI: −8.97 to −6.44). A total of 399 (24.6%) individuals with DS were considered vitamin D deficient, compared to 1472 (28.3%) with ASD and 12,397 (40.3%) controls (*p* < 0.001, 95% CI: −5.43 to −2.36).

The impact of demographic and clinical characteristics on mean 25(OH)D levels in individuals with DS is presented in Table 2. Multivariate linear analysis revealed that older individuals had greater levels of vitamin D 25-OH (0.18 ng/mL/year, *p* < 0.05, 95% CI: 0.04–0.32). Individuals with DS with higher BMI were found to have lower mean levels of 25(OH)D (−0.27 ng/mL/kg/m^2^, *p* < 0.001, 95% CI: −0.3849 to −0.1509). Additionally, individuals with a neurologic diagnosis have decreased 25(OH)D levels compared to those without neurological disorders (−3.15 ng/mL, *p* < 0.001, 95% CI: −5.02 to −1.28). Although not statistically significant, Native American individuals had higher mean 25(OH)D levels compared to Caucasians (2.81 ng/mL, 95% CI: −0.492–10.55), whereas Asian, Black, and other races had lower mean levels than Caucasians (−0.17, −1.63, and −1.85 ng/mL, respectively). Individuals with DS and autoimmune disease had lower 25(OH)D levels (−3.11 ng/mL, *p* < 0.001, 95% CI: −6.22 to −1.55). Similarly, a history of autoimmunity in a first-degree relative also is associated with lower levels of 25(OH)D in persons with DS (−1.83 ng/mL, *p* = 0.01, 95% CI: −2.45 to −0.63). Hyperthyroidism, obstructive sleep apnea, congenital heart disease, sex, Hispanic or non-Hispanic ethnicity, having both DS and ASD, and restricted dietary intake did not have a statistically significant association with 25(OH)D levels in individuals with DS.

**Table 2:** Impact of demographic and clinical characteristics on vitamin D status in individuals with Down syndrome

	**Adjusted mean difference (ng/mL)**	**95% CI**
**Age (years)**	0.18	0.04–0.31*
**Sex**	−0.04	−1.23–1.14
**BMI (kg/m**^**2**^**)**	−0.27	−0.38 to −0.15***
**Race**		
*Caucasian (RC)*	--	--
*Asian*	−0.17	−2.17–1.83
*Black*	−1.63	−4.33–1.06
*Native American*	2.81	−4.92–10.55
*Other*	−1.85	−8.23–4.53
*Unknown*	22.42	8.74–36.11**
**Ethnicity**		
*Hispanic (RC)*	--	--
*Not Hispanic*	0.32	−0.91–1.54
**Autism spectrum disorder**		
*Present (RC)*	--	--
*Not present*	−0.09	−1.79–1.61
*Unknown*	2.44	−2.31–7.19
**Personal autoimmune disease**	−3.11	−6.22 to −1.55***
**1st-degree family with autoimmune disease**	−1.83	−2.45 to −0.63**
**Hypothyroidism**		
*Present (RC)*	--	--
*Not present*	0.89	−0.96–2.74
*Unknown*	1.04	−0.94–3.02
**Obstructive sleep apnea**		
*Present (RC)*	--	--
*Not present*	0.79	−0.96–2.74
*Unknown*	0.73	−0.94–3.02
**Congenital heart disease**		
*Present (RC)*	--	--
*Not present*	−0.18	−1.88–1.52
*Unknown*	3.59	−20.23–27.42
**Neurologic disease**	−3.15	−5.02 to −1.28***
**Restricted dietary intake or failure to thrive**		
*Present (RC)*	--	--
*Not present*	−1.15	−3.11–0.80
*Unknown*	−1.16	−3.47–1.14

*RC* Reference category, *CI* Confidence interval

^***^p < 0.001, ^**^p < 0.01, ^*^p < 0.05

Associations between demographics and 25(OH)D levels in the cohort of individuals with ASD are presented in Table 3. Multivariate linear regression analysis identified that older individuals with ASD had lower mean 25(OH)D levels (−0.19 ng/mL/year, *p* < 0.001, 95% CI: −0.26 to −0.12). Individuals with a reported “unknown ethnicity” had higher mean levels of 25(OH)D than Hispanic ethnicity (reference category) (2.69 ng/mL, *p* = 0.01, 95% CI: 0.93–4.44). Race, BMI, and sex did not have a significant impact on mean 25(OH)D levels. Like the ASD cohort, controls had lower 25(OH)D levels at an older age (−0.39 ng/mL/year, *p* < 0.001, 95% CI: −0.45 to −0.33), as shown in Table 3.

**Table 3:** Adjusted mean 25(OH)D level difference in children with ASD and neither ASD nor DS

	**Adjusted mean difference on 25(OH)D level (95% CI) ASD cohort (ng/mL)**	**Adjusted mean difference on 25(OH)D level (95% CI) neurotypical control (ng/mL)**
**Age (years)**	−0.19 (−0.26–0.12)***	−0.39 (−0.45 to −0.33)***
**BMI (kg/m**^**2**^**)**	0.004 (−0.006–0.014)	−0.003 (−0.02–0.01)
**Sex**	0.23 (−0.51–0.98)	−0.56 (−1.21–0.08)
**Race**		
*Caucasian (RC)*	--	--
*Asian*	0.44 (−2.0–1.12)	−1.82 (−3.31 to −0.33)*
*Black*	1.41 (−0.94–3.75)	0.79 (−1.24–2.82)
*Nat. Amer.*	−1.97 (−25.46–21.52)	-
*Other*	−0.21 (−1.14–0.72)	−0.01 (−0.79–0.77)
*Unknown*	−0.37 (−2.24–1.49)	−0.30 (−1.87–1.27)
**Ethnicity**		
*Hispanic (RC)*	--	--
*Not Hispanic*	0.60 (−0.32–1.52)	2.10 (1.29–2.91)***
*Unknown*	2.69 (0.93–4.44)**	0.36 (−1.10–1.82)

*CI* Confidence interval, *Nat. Amer* Native American, *RC* Reference category

^***^p < 0.001, ^**^p < 0.01, ^*^p < 0.05

**Fig. 2:**
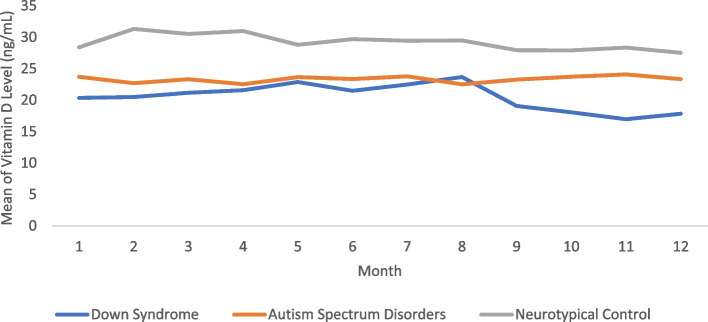
Temporal trends in 25(OH)D status

Individuals with DS and ASD have lower variability in 25(OH)D levels across the year than the individuals in the control group. The cohort with DS had a standard deviation of 12.39 ng/mL, and the cohort with ASD had a standard deviation of 11.96 ng/mL. The controls had more than twice the variability of DS and ASD at 27.43 ng/mL. Figure 2 illustrates the 25(OH)D levels for all three cohorts from January to December. Overall, levels for all three groups remain steady with the group with DS slightly decreasing in levels in September (month 9) through December (month 12).

Across all groups, individuals of Hispanic ethnicity had lower mean 25(OH)D levels as compared to those with non-Hispanic ethnicity (−2.16 ng/mL, *p* < .001, 95% CI: −2.68 to −1.64). Multivariate analysis of race did not have a statistically significant correlation with individual’s 25(OH)D levels (*p* = 0.17 95% CI: −8.97–0.35).

## Discussion

In this large, multi-center study of vitamin D status, individuals with DS were observed to have lower 25(OH)D levels in comparison to individuals with ASD and NT controls. This finding is consistent with previous, smaller studies and provides high-quality multi-center data confirming these findings in a racially and neurodevelopmentally diverse cohort [5, 7, 18–21]. To our knowledge, this is the largest study of vitamin D status in individuals with DS. In addition, findings are strengthened by the utilization of data from sites in three unique geographical locations in the USA.

We found a statistically significant association between low vitamin D and the presence of autoimmune disease. In our DS cohort, both personal and family history of autoimmune disorder were highly associated with lower levels of vitamin D. Multiple prior studies have linked autoimmune disorders in individuals with DS and hypovitaminosis D [6, 22–26]. As vitamin D plays an immunoregulatory role, deficiency may lead to the development of autoimmunity, although this study was not designed or powered to assess causality [27–31]. However, the higher rates of hypovitaminosis D in individuals with DS and autoimmune disorders could provide a potential therapeutic target for this population to either prevent or adjunctly treat autoimmune disorders. Vitamin D is crucial for immune regulation as its receptors are expressed in various immune cells, including T-cells and dendritic cells [32–35]. Vitamin D assists in modulating T-cell proliferation and promoting anti-inflammatory responses in the brain [36, 37]. As a result, hypovitaminosis D has been causally linked to a wide variety of autoimmune diseases, some of which have neurologic involvement, as well such as multiple sclerosis [38, 39]. This is particularly important given the dual association of high rates of hypovitaminosis D and autoimmunity in persons with DS, although the exact mechanism of this relationship remains unknown [3, 40, 41]. An exciting next step from this study would be to prospectively assess rates of development of autoimmune diseases in individuals with DS who use vitamin D supplementation and those who do not.

Interestingly, race and ethnicity did not significantly impact 25(OH)D levels in this study, in contrast with prior findings of higher rates of hypovitaminosis D in Black and Asian individuals and those of Hispanic ethnicity [42–44]. In our study, Hispanic ethnicity was associated with higher 25(OH)D levels in the control group, although this may be secondary to the fact that a majority of Hispanic participants in the study resided in more temperate climates; since the main source of vitamin D is skin exposure to UVB radiation from the sun, study site location likely explains these observations [36].

Among individuals with DS, older age was associated with higher levels of vitamin D. This association was opposite that of individuals with ASD and NT controls, where older age was associated with lower levels of 25(OH)D. Persons with DS experience developmental milestone delays, which may result in decreased outdoor activity at younger ages, reducing natural vitamin D production from skin exposure to UVB radiation from the sun. Sex did not have a statistically significant impact on 25(OH)D levels in individuals with ASD, DS, or NT controls. Although sex-based differences in vitamin D have been observed in prior studies with conflicting results, such differences may not be present in our cohort due to the high number of pre-pubescent individuals evaluated (median age: 13) [42, 45].

Various clinical factors may account for the observed differences in 25(OH)D levels within our cohort with DS. Higher BMI is more common in individuals with DS [46, 47], as was found in our study and was associated with lower levels of 25(OH)D among individuals with DS. Higher BMI is associated with increased body fat, which can also sequester vitamin D, which may provide an explanation for this finding [48, 49]. In our study, restricted dietary intake did not impact 25(OH)D levels in persons with DS, although this was an a priori concern given reports of restricted eating and textural sensitivity in this population [50–52]. This would have been a similar concern in individuals with ASD, but this was not frequently reported in the clinical documentation for this population. A consideration for why this was not observed is that this population may be more likely to receive nutritional supplementation although this was not assessed in this study.

Individuals with both DS and ASD have been shown to have lower 25(OH)D levels compared to NT controls in previous research studying ASD or DS alone [5, 7, 18–21], but the findings we present show mean 25(OH)D levels in persons with DS are lower than those with ASD. Deficiency in 25(OH)D can potentially hinder typical neural proliferation processes and could potentially impact long-term developmental outcomes [36]. However, supplementation of vitamin D in individuals with ASD has inconsistently yielded any health or cognitive benefits [16, 53–56]. The genotypic and phenotypic heterogeneity in the ASD population, the age at which vitamin D deficiency is identified, as well as timing of supplementation may all contribute to the variability in reports of therapeutic benefit to vitamin D replacement for individuals with ASD. This may yield some variability in reports of therapeutic benefit although the timing of supplementation and age at identification are also likely to be modifiers in this condition.

An area of future investigation will be to determine if DS itself (specifically trisomy of chromosome 21) may have some role in the etiology of this lower 25(OH)D level. Emerging evidence may place interferon drive dysregulation as a possible modifying mechanism for vitamin D homeostasis, although this has not been directly studied [57–59]. In addition, further investigation will need to address factors such as baseline physical activity, mobility, median time outdoors, and likelihood of nutritional supplementation, all of which could impact the prevalence of hypovitaminosis D in this population [60, 61].

This study is not without limitations. This study is retrospective in nature and limited clinical data or rationale for 25(OH)D testing was ascertained from existing records. As such, there are many unknowns regarding the individual participants, including supplementation status amount of exposure to UVB radiation, exercise, and dietary habits, all of which affect 25(OH)D levels. While these are significant limitations, the large, multi-center, multi-year cohort design of this study strengthens thematic trends noted with regard to hypovitaminosis D in these populations. As the three primary centers involved in this study were tertiary academic medical centers with DS programs, there is likely to be severity bias within the cohorts as they do not represent classic community sampling. This would presumably lead to a higher likelihood of reporting hypovitaminosis D in all groups. Despite the statistically significant finding of hypovitaminosis D in individuals with both DS and ASD, lower levels of 25(OH)D did not correlate with increased incidence of vitamin D deficiency, with NT controls experiencing the highest incidence of vitamin D deficiency, followed by individuals with ASD, then persons with DS. This may be an artifact of the spread of the data, in which the interquartile range of our DS cohort was substantially lower than both the ASD and control cohorts.

In summary, individuals with DS were noted to have hypovitaminosis D in comparison to individuals with ASD and NT controls. In individuals with DS, hypovitaminosis D was also correlated with the presence of auto-immune disease. These findings warrant further investigations into the etiology of vitamin D deficiency in individuals with DS, and into the impact of supplementation, particularly as it pertains to the prevention of autoimmune disease across the lifespan.

### Supplementary Information

**Additional file 1: Supplement 1.** ICD-9 and ICD-10 codes for neurotypical controls.
