# Supplementary material for: Hypovitaminosis D in persons with Down syndrome and autism spectrum disorder
Source: J Neurodev Disord. 2023 Oct 25;15:35. doi: 10.1186/s11689-023-09503-y (PMC10599027; doi:10.1186/s11689-023-09503-y)
Supplement: Supplementary file 1 — Additional file 1: Supplement 1. ICD-9 and ICD-10 codes for neurotypical controls. [file 11689_2023_9503_MOESM1_ESM.docx]

**Supplement 1:** ICD-9 and ICD-10 codes for neurotypical controls.

| *ICD-9 Codes* | |
| --- | --- |
| V20.2 | Routine infant or child health check |
| V20.31 | Health supervision for newborn under 8 days old |
| V20.32 | Health supervision of newborn 8 to 28 days old |
| V70 | Routine general medical examination at a health care facility |
| V70.5 | Health examination of defined subpopulations |
| V70.6 | Health examination in population surveys |
| V72.83 | Pre-procedural general physical examination |
| V73 | Special screening examination for other specified viral diseases |
| *ICD-10 Codes* | |
| Z00.0 | Encounter for general adult medical examination |
| Z00.129 | Encounter for routine child health examination |
| Z00.8 | Encounter for other general examination |
| Z01.0 | Encounter for examination of eyes and vision |
| Z01.81 | Encounter for preprocedural examinations |
| Z02.89 | Encounter for other administrative examinations |
| Z71.84 | Encounter for health counseling related to travel |
| Z13.3 | Encounter for screening examination for mental health and behavioral disorders |
